# DNA Barcoding of genus *Hexacentrus* in China reveals cryptic diversity within *Hexacentrus japonicus* (Orthoptera, Tettigoniidae)

**DOI:** 10.3897/zookeys.596.8669

**Published:** 2016-06-07

**Authors:** Hui-Fang Guo, Bei Guan, Fu-Ming Shi, Zhi-Jun Zhou

**Affiliations:** 1 The Key Laboratory of Zoological Systematics and Application, College of Life Sciences, Hebei University, Baoding 071002, China

**Keywords:** BOLD, China, DNA Barcoding, Hexacentrus, species delineation

## Abstract

DNA barcoding has been proved successful to provide resolution beyond the boundaries of morphological information. Hence, a study was undertaken to establish DNA barcodes for all morphologically determined *Hexacentrus* species in China collections. In total, 83 specimens of five *Hexacentrus* species were barcoded using standard mitochondrial cytochrome c oxidase subunit I (COI) gene. Except for *Hexacentrus japonicus*, barcode gaps were present in the remaining *Hexacentrus* species. Taxon ID tree generated seven BOLD’s barcode index numbers (BINs), four of which were in agreement with the morphological species. For *Hexacentrus japonicus*, the maximum intraspecific divergence (4.43%) produced a minimal overlap (0.64%), and 19 specimens were divided into three different BINs. There may be cryptic species within the current *Hexacentrus japonicus*. This study adds to a growing body of DNA barcodes that have become available for katydids, and shows that a DNA barcoding approach enables the identification of known *Hexacentrus* species with a very high resolution.

## Introduction

DNA barcoding employs short, standardized gene regions (5' segment of mitochondrial cytochrome oxidase subunit I for animals) as an internal tag to enable metazoan species identification (Hebert et al. 2003). Schmidt et al. (2015) found that DNA barcoding largely supported 250 years of classical taxonomy for central European bees. Unlike distinct species, closely related species offer a great challenge for phylogeny reconstruction and species identification with DNA barcoding due to overlapping genetic variation (Dai et al. 2012). For example, Versteirt et al. (2015) found that DNA barcoding offered a reliable framework for mosquito species identification in Belgium except for some closely related species. Zhou et al. (2012) found that molecular identification with DNA barcoding supported most traditional morphological species of genus *Ruspolia* in China.

In this study, our objective is to assess the utility of DNA barcoding for closely related katydid species, belonging to the genus *Hexacentrus* (Serville, 1831) in China. *Hexacentrus* is mainly distributed in Australian, Afrotropical and Oriental realms. *Hexacentrus* is a particularly speciose genus, containing 24 known species (Eades et al. 2016). *Hexacentrus* was the single genus within Hexacentrinae, which has been reported in China according to “Orthoptera Species File” (Eades et al. 2016). Up to now, a total of six *Hexacentrus* species have been reported, including *Hexacentrus japonicus* (Karny, 1907), *Hexacentrus unicolor* (Serville, 1831), *Hexacentrus yunnaneus* (Bey-Bienko, 1962), *Hexacentrus fuscipes* (Matsumura & Shiraki, 1908), *Hexacentrus mundus* (Walker, 1869) and *Hexacentrus expansus* (Wang and Shi 2005). Due to the rather difficult morphological discrimination between *Hexacentrus* species, an interspecific molecular delineation was needed. To make *Hexacentrus* more accessible to the scientific community, the open access project “BHC” had been initiated in the Barcoding of Life Data systems (BOLD) (Ratnasingham and Hebert 2007). There are a few DNA barcoding study concentrated on *Hexacentrus*, and the number of barcode sequences was limited in BOLD. The goals of this study are as following: (i) it will allow scientists with molecular capability but insufficient knowledge of *Hexacentrus* taxonomy and systematics to recognize species and document the biodiversity of *Hexacentrus*. (ii) For Tettigoniidae taxonomists, it contributes to integrative taxonomic approaches, such as the elucidation of related species and clarification of problematic species groups, association of the sexes within one species, and the identification of new species (Gibbs 2009, 2011, Packer et al. 2009, Schmidt et al. 2015). To this end, we checked for the presence of species barcode gaps and cryptic diversity within species. BOLD’s barcode index number (BIN) analysis tool (Ratnasingham and Hebert 2013) was used to analyze *Hexacentrus*.

## Material and methods

### Collection of specimens

All specimens were collected by hand or sweeping method during their active season (July–November). *Hexacentrus* species were all gathered in China from 12 localities, with the latitude from 18.70°N to 41.80°N and the longitude from 97.83°E to 123.38°E. One or more specimens were chosen from each locality in order to include as many morphologically distinguishable individuals per site as possible. Specimens were collected and stored in 100% ethanol at -20 °C and were deposited in the Hebei University Museum. Species-level identification was based on the original morphological descriptions, locality data and additional information. Details on all specimens (sampling location, GPS coordinates, voucher number, BOLD number, etc.) are available within the “DNA Barcoding of *Hexacentrus* in China, BHC” project in the Barcode of Life Data Systems (BOLD. www.barcodinglife.org).

### DNA extraction, amplification and sequencing

Total DNA was extracted from the muscle of one hind leg of each specimens using TIANamp Genomic DNA Kit in accordance with the manufacturer’s instructions. The standardized gene regions of animals DNA barcoding was amplified using the primers COBU (5'-TYT CAA CAA AYC AYA ARG ATA TTG G-3') and COBL (5'-TAA ACT TCW GGR TGW CCA AAR AAT CA-3') (Pan et al. 2006). The 50 μL polymerase chain reaction (PCR) mixture contained 3 μL of template DNA, 5 μL of 10 × buffer, 4 μL of dNTP mix, 5 μL of each primer (10 μM each), 0.5 μL of *Taq* polymerase (5 U/μL), and 27.5 μL of water. The thermal profile was: 94 °C for 3 min, 34 cycles at 94 °C for 30 s, 49 °C for 30 s, and 72 °C for 90 s, and final extension at 72 °C for 8 min. PCR products were visualized in 1% agarose gels electrophoresis. PCR products were sequenced directly using ABI BigDye Terminator chemistry on ABI3730 automated sequencer (Applied Biosystems) in Genewiz Inc. (Beijing, China), and in both directions to minimize PCR artifacts, ambiguities and base calling error.

### Data analysis

Consensus sequence of both directions was assembled using SeqMan in Lasergene and verification of ambiguities and unexpected stop codons were performed in EditSeq (Burland 2000). Sequence alignments were conducted using Clustal X 1.81 (Thompson et al. 1997) with default parameters. The both ends of the sequences matching the primer sequences were excised to remove artificial nucleotide similarity introduced by PCR amplification, resulting in the final data sets for barcoding analysis.

The analyses were restricted to the subset of sequences, which met barcode standards (sequence length > 500bp, < 1% ambiguous bases, bidirectional sequencing, country specification). Intra- and inter-specific genetic distances were based on the Kimura-2-parameter (K2P) model (Kimura 1980) using the ‘distance summary’ tool in BOLD. The barcode gap was defined by intraspecific vs. interspecific [nearest neighbor (NN)] genetic distance of species. A globally unique identifier (i.e. BIN) then was assigned to each sequence cluster, creating an interim taxonomic system because the members of a particular BIN often correspond to a biological species. Character based DNA barcoding used the nucleotide variation in each position across DNA regions as diagnostic characters.

## Results

COI sequences were recovered from 86 of the 91 specimens that were analyzed with barcode compliant records from 83 specimens representing five species. Three records have no barcode compliant records because of low quality of trace file. A number of 80 barcodes belong to four previously identified species whereas three analyzed specimens were only identified to genus level because they are female; they probably are *Hexacentrus yunnaneus* due to collection in Yunnan and separated from other specimens. The 658bp length sequences without indel (insertion/deletion) had full-length records. COI sequences were translated to amino acid sequences to check for stop codons and shifts in reading frame that might indicate the presence of nuclear mitochondrial copies (numts), but none were detected. Diagnostic character analysis was consistent with that of traditional external appearance discrimination. *Hexacentrus expansus* only having one specimen was not analyzed, thus lacking diagnostic character.

### Distance summary and Barcode Gap analysis

Mean intraspecies divergence was 1.32% (ranged between 0.57% and 2.43%), and maximum intraspecies divergence 4.43% was observed in *Hexacentrus japonicus* (Table 1). When correcting for the uneven sample sizes of species, the within-species divergence decreased from 1.32% to 1.23%. Between *Hexacentrus* species, the average K2P genetic distance was 12.54%, whereas minimum genetic distance only 3.79% (Table 1).

**Table 1.** Mean and maximum intraspecific and nearest neighbor (NN) distance for all specimens.

Species	Mean intraspecific distance	Max intraspecific distance	Nearest neighbor	Distance to NN
*Hexacentrus expansus*	N/A	N/A	*Hexacentrus unicolor*	13.19
*Hexacentrus japonicus*	2.43	4.43	*Hexacentrus mundus*	3.79
*Hexacentrus mundus*	0.57	0.93	*Hexacentrus japonicus*	3.79
*Hexacentrus* sp.	0.72	1.08	*Hexacentrus unicolor*	9.72
*Hexacentrus unicolor*	1.19	3.79	*Hexacentrus* sp.	9.72

**Figure 1.**
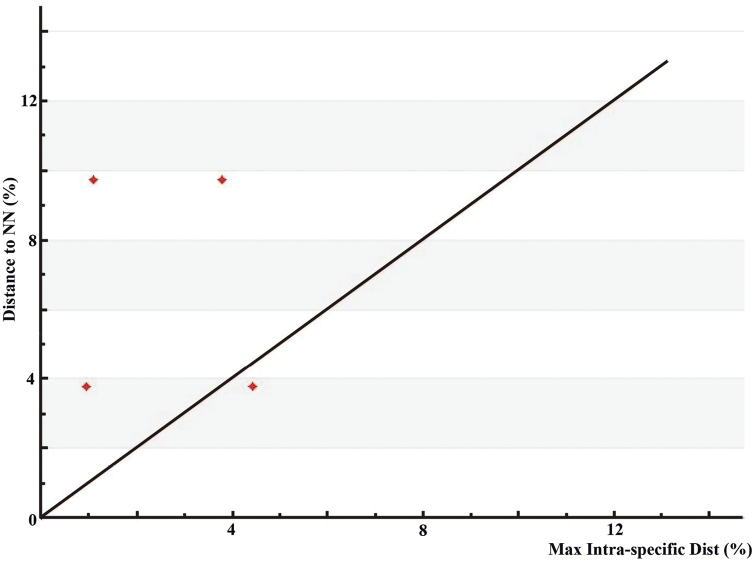
Barcode gap plot showed the distance to the nearest neighbor (NN) vs. the maximum intraspecific distance Kimura-2-parameter (K2P) for 83 specimens. Dots above the 1:1 line indicated the presence of a barcode gap.

Singleton species (*Hexacentrus expansus*) were excluded from barcode gap analysis. Except for *Hexacentrus japonicus*, barcode gap was present in the remaining *Hexacentrus* species (Fig. 1). Although the maximum intraspecific divergence of *Hexacentrus unicolor* was more than 2% (ranged between 0 and 3.79%), but still less than minimum interspecific between *Hexacentrus unicolor* and its NN (Nearest Neighbor) *Hexacentrus* spp. However, the maximum intraspecific divergence of *Hexacentrus japonicus* (4.43%) produced a minimal overlap (0.64%).

### Taxon ID tree analysis

**Figure 2.**
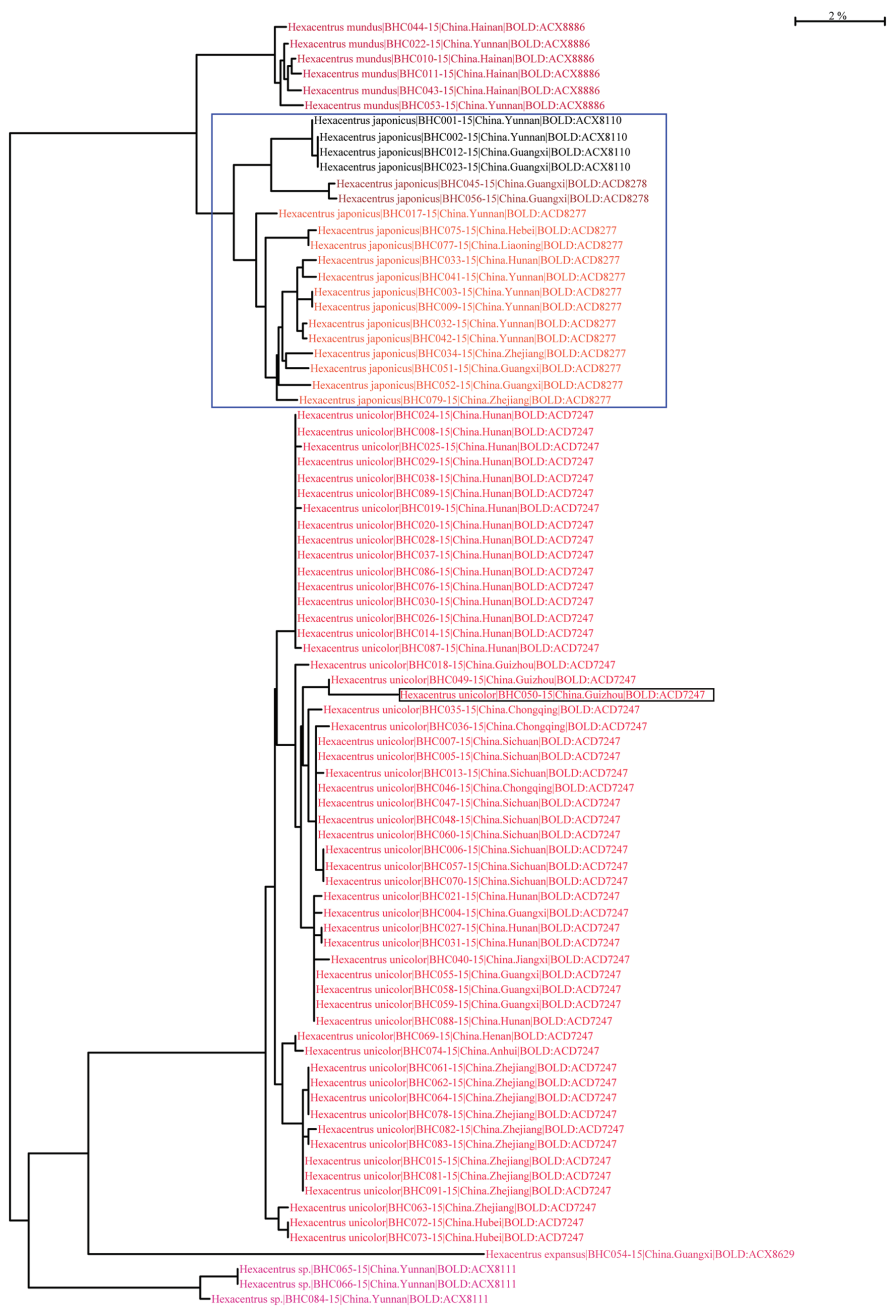
Taxon ID tree revealed seven well-differentiated haplogroups. Process ID, location, and BINs were shown in the tree. The clusters with a blue box indicated there may be two new putative ‘cryptic species’ within *Hexacentrus japonicus*. The clade with a black box indicated the specimen had more mutation within *Hexacentrus unicolor*.

The taxon ID tree was divided into seven clades represented by different BINs (Fig. 2). *Hexacentrus unicolor*, *Hexacentrus mundus* and *Hexacentrus expansus* were composed well-supported monophyletic groups, which were fully congruent with the morphological species. *Hexacentrus japonicus* were divided into three different BINs. Although no morphological differences were observed among these three BINs, there might be cryptic species within the current *Hexacentrus japonicus*.

### Diagnostic characters analysis

Forty-four diagnostic characters were found in the study (Table 2). Four *Hexacentrus* species had diagnostic characters and the success rate was 80%. The number of diagnostic character sites of *Hexacentrus* spp. was no less than 20 (Table 2), which may be caused by scarce specimens and by the relative distance of the phylogenetic relationship to others.

**Table 2.** Character-based DNA barcodes for four *Hexacentrus* species of COI gene. A_7* means A is at the 7th position.

Species	Diagnostic characters	Characters no.	Specimen no.
*Hexacentrus* sp.	A_7* G_184 C_247 A_301 A_304 T_346 G_391 C_400 G_424 C_463 C_472 C_500 T_517 G_550 G_586 C_607 A_619 C_622 C_625 G_628	20	3
*Hexacentrus unicolor*	T_25 T_136 T_223 A_227 T_322 T_379 T_424 G_487 A_502 C_517 T_529 C_530 G_532 C_550 C_586 G_619 T_631	17	54
*Hexacentrus mundus*	T_34 T_118 C_187 T_397 A_643	5	6
*Hexacentrus japonicus*	T_460 C_514	2	19

## Discussion

The present study evaluated the efficacy of using DNA barcodes for the identification of *Hexacentrus* in China and provided a group of sequences associated with the identified species. Using these DNA barcoding, not only can one delineate the boundaries between species, but also assign taxonomic status to unknown specimens from known species.

*Hexacentrus unicolor* was controversial, and *Hexacentrus plantaris* (Burmeister, 1838) and *Tedla sellata* (Walker, 1869) were considered as its synonyms. *Hexacentrus unicolor* is distributed in south of the Yangtze River. In this study, however, one specimen was collected from Henan. In fact, molecular data support *Hexacentrus unicolor* as a single group, with all specimens sharing one BIN (BOLD: ACD 7247). The specimen with black box (Fig. 2) came from Guizhou, and had more mutations compared to the other *Hexacentrus unicolor* specimens. We cannot be completely certain that this phenomenon was due to geographic isolation rather than sequencing or calibrating errors. *Hexacentrus japonicus* was closely related to *Hexacentrus unicolor*, and they are widely distributed in the southwest, central and south areas of China. The tegmina of male *Hexacentrus japonicas* was short and broad (about 2.75–3.00 times), whereas *Hexacentrus unicolor* was long and narrow (about 2.95–3.30 times) as long as broad (Wang and Shi 2005). Nevertheless, the analyses revealed that *Hexacentrus japonicus* ranged to Hebei and Liaoning. Interestingly, *Hexacentrus japonicus* contained three BINs (BOLD: ACX 8110, BOLD: ACD 8277, BOLD: ACD 8278) with high intraspecific distance (> 2%). This group included 20 specimens, in which no morphological differences were found. Therefore it is necessary to clarify the status of this species complex as it may include more species than currently recognized. *Hexacentrus mundus* was only recorded in Guangxi and Yunnan. In this study, four specimens from Hainan were also identified as *Hexacentrus mundus* because only 2–3 larger teeth in the middle part of stridulatory file, however there are 6–7 large teeth in *Hexacentrus japonicas* and *Hexacentrus unicolor* (Wang and Shi 2005). Thus *Hexacentrus mundus*’s distribution was enlarged. All specimens of *Hexacentrus mundus* were assigned as 1 BIN, which clearly confirm a consistency between molecular and morphological analyses. *Hexacentrus expansus*, due to the obviously inflated male tegmina, was easy to identify only by the morphological method. BIN assignments also revealed that *Hexacentrus expansus* was a separated clade with only a single male available for analysis. The specimens from Yunnan almost certainly represent the ‘true’ *Hexacentrus yunnaneus* because the type location of this species (Hekou, Yunnan) was in close proximity. Hence, further specimens are needed to be analyzed, especially male material.

DNA barcoding, as one effective tool in insect taxonomy, had been already applied widely. It can rapidly acquire molecular data, simplifying species classification and identification. Yet, DNA barcodes has been argued to be unreliable for consistent species identification by many authors due to a number of drawbacks (DeSalle et al. 2005; Will et al. 2005; Rubinoff et al. 2006; Ebach 2011). Recent speciation, incomplete lineage sorting, interspecific hybridization and infection by endosymbiotic bacteria such as *Wolbachia* (Funk and Omland 2003) may all interfere with the performance of DNA barcoding in insects (Virgilio et al. 2010). In this context, most of the species can be amplified successfully; however, five specimens cannot be translated and three specimens only had one represented trace file, which cannot meet the DNA barcoding standards. The deep inspection of trace files indicated that most of these failures arose from co-amplification of the bacterial endosymbiont *Wolbachia*, which disturbed normal interpretation of trace file. It had been estimated that *Wolbachia* is present in two-thirds of all insect species (Hilgenboecker et al. 2008). There was no reason to doubt the absence of *Wolbachia* in *Hexacentrus*. Zhou et al. (2014) found that the nuclear sequence of mitochondrial (numts) reported in *Mecopoda niponensis* may form a separate clade. The same case was reported in *Podisma pedestris* (Bensasson et al. 2000). But in this study, the clades in *Hexacentrus japonicus* were not caused by numts for all sequences analyzed without translation early termination, base indel, frameshift mutations. On the other hand, geographic isolation was also rejected, for only the specimens from Yunnan included three BINs in this group. The most probable reason was the existence of cryptic species compared to other *Hexacentrus* species.

Species boundaries were hard to delimit only based on morphologyas, and analyses including additional sources of information such as molecular data, biogeography, behavior and ecology has been called integrative taxonomy which has been shown to be very useful (Dayrat 2005). We are convinced that DNA barcoding can promote the *Hexacentrus* species identification. Our study showed that all known *Hexacentrus* species could be delimitating rapidly through DNA barcoding in China, except for *Hexacentrus japonicus*. *Hexacentrus japonicus* was problematic when using BOLD analysis. Finally, regardless of the promising results, the incorporation of nuclear genes is valuable for species delimitation and might strengthen the results, as they are independent of the maternal inherited mitochondrial genes.

The ideal situation would be that each species was represented by sequence from its type material, particularly the holotype. Type specimens were also dried specimens and DNA degraded at different level, so not only amplification was difficult, but also the damage of specimens can’t be neglected. Recently, Prosser et al. (2016) successfully obtained sequences from century-old type specimens using next-generation sequencing (NGS). We believed that DNA barcoding is useful in revealing cryptic biodiversity, potentially facilitating traditional taxonomy in future.
